# The impact of changes in leisure time physical activity on changes in cardiovascular risk factors: results from The Finnmark 3 Study and SAMINOR 1, 1987–2003

**DOI:** 10.1080/22423982.2018.1459145

**Published:** 2018-04-15

**Authors:** Rune Hermansen, Ann Ragnhild Broderstad, Bjarne K. Jacobsen, Markku Mähönen, Tom Wilsgaard, Bente Morseth

**Affiliations:** a Department of Community Medicine, UiT The Arctic University of Norway, Tromsø, Norway; b Finnmark Hospital Trust, Kirkenes Hospital, Kirkenes, Norway; c Centre for Sámi Health Research, Department of Community Medicine, UiT The Arctic University of Norway, Tromsø, Norway; d Faculty of Medicine, University of Oulu, Oulu, Finland; e School of Sport Sciences, UiT The Arctic University of Norway, Tromsø, Norway

**Keywords:** Physical activity, ethnicity, cardiovascular risk factors, longitudinal, Sami

## Abstract

**Objective**: The aim of this study was to examine the associations between changes in leisure time physical activity and changes in cardiovascular risk factors over 16 years and whether they differ between two ethnic groups in Norway.

**Methods**: Data were extracted from two population-based studies. Altogether, 3671 men and women participated in both surveys, and 30% reported being of Sami ethnicity. Leisure time physical activity was self-reported, and cardiovascular risk factors were measured. ANCOVA analysis was used to examine associations between changes in physical activity and changes in cardiovascular risk factors.

**Results**: After adjustment for age, sex, smoking, ethnicity and respective baseline values, favourable changes in body mass index (BMI) and levels of triglycerides were most pronounced in those who were active in both surveys (*p* < 0.05) whereas the opposite was the situation for cholesterol levels (*p* = 0.003). Changes in systolic blood pressure, diastolic blood pressure and resting heart rate were not significantly associated with change in physical activity. Ethnicity did not influence the associations between physical activity and cardiovascular risk factors.

**Conclusion**: Traditional cardiovascular risk factors were to a small extent associated with change in leisure time physical activity. Persistent physical activity was associated with beneficial changes in BMI and triglycerides.

## Introduction

A sedentary lifestyle is considered a health risk, and physical activity is an important modifiable factor contributing to risk reduction of cardiovascular diseases (CVD) and death [1–3]. Although the incidence of CVD in Norway has declined over the last decades [4], CVD is still a major cause of premature death and morbidity, and focus on preventive measures such as physical activity should continue [5–7].

Although numerous intervention studies suggest beneficial effects of physical activity on cardiovascular risk [8], the majority of studies are restricted to a single point measure of physical activity. The relatively few studies that have included repeated measurements of leisure time physical activity indicate that increased physical activity is associated with improved glucose, insulin and lipid metabolism and may prevent or delay the onset of metabolic syndrome [9,10]. Due to the scarcity of studies concerning change in physical activity and CVD risk factors, and because many individuals change their behaviour over time [11], we wanted to address this issue in a cohort of Norwegian adults of different ethnic origin with repeated measurements of physical activity.

The main aim of this study was to examine the association between changes in leisure time physical activity and changes in cardiovascular risk factors. Additionally, we aimed to examine whether the relationship between change in leisure time physical activity and change in cardiovascular risk factors differs between two ethnic groups in Norway.

## Methods

### Study population

#### Baseline population: the Finnmark 3 Study

The Finnmark Study is a population-based study of cardiovascular risk factors and disease in Finnmark County. The study started in 1974 and was conducted by the National Health Screening Service in collaboration with the University of Tromsø and local health authorities. The study design included repeated population health surveys, to which total birth cohorts and samples were invited. The present analyses are based on data from the third Finnmark Study in 1987–1988. The study was approved by The Norwegian Data Inspectorate.

All resident men and women aged 40–62 years were invited to Finnmark 3. In addition, subjects aged 20–39 years, who had been invited to the second survey in 1977 and were still living in Finnmark, together with a 10% random sample of men and women in the same age group, were also invited. A total of 17,864 men and women attended Finnmark 3, representing 78% attendance rate. Briefly, all subjects were invited by a personal letter to Finnmark 3, which included a one-page questionnaire. In four municipalities with a high proportion of Sami inhabitants, the questionnaire was available in both Sami and Norwegian language. Family history of coronary heart disease, prevalent CVD and diabetes, level of physical activity at work and during leisure time, ethnicity, and smoking habits were covered. The questionnaire was completed at home and checked by trained nurses for inconsistencies at the clinical examination. Further details on the study design have been published previously [12,13].

#### Follow-up population: the SAMINOR 1 Study

SAMINOR is a study of health and living conditions in areas in Norway with Sami and Norwegian settlement. The indigenous Sami population is a minority living in the northern part of Norway, Finland, Sweden and Russia’s Kola Peninsula. Traditionally, a relatively high proportion of the Samis have been working with agriculture and reindeer herding. SAMINOR 1 was conducted in 2003–2004 in municipalities in Norway with more than 5–10% of the population reported to be Sami in the 1970 Census [14]. In total, 24 municipalities were included, of which nine were located in the county of Finnmark. All areas had mixed Sami and non-Sami populations [15]. The SAMINOR 1 Study included questionnaires and a basic clinical examination. Details concerning screening procedures and methods have been published previously [15]. Briefly, all inhabitants from the preselected municipalities aged 30 and 36–79 years were invited. Subjects who were invited to the cardiovascular screening program in Finnmark in 1987–1988 were also included in the SAMINOR cohort. The questionnaire focused on living conditions, health, Sami traditions and ethnicity. The self-administrated questionnaire was designed to extract information regarding chronic lifestyle diseases, physical activities, smoking habits and diet, in addition to questions such as age, sex, education and marital status. Information about ethnicity was based on a combination of questions on self-identification and language used at home. The questionnaire and the informed consent forms were available in Sami and Norwegian languages. The SAMINOR 1 Study was accredited by the Regional Board of Research Ethics in Northern Norway and by the Board’s Sami Consultant. The survey is in accordance with the Helsinki Declaration of 1975. The National Data Protection Authority approved the use of personal information, and the study is registered with the number 2002/1525–2. A total of 16,489 men and women who were residents of Finnmark were invited to SAMINOR 1, and 10,411 subjects (63.1%) participated in the study. Ethnicity was known for 10,170 subjects, and 4346 participants reported Sami affiliation (42.7%) [15].

#### Selected sample for the present analyses

The cohort included in the present analyses consisted of participants from the 1987–1988 survey (Finnmark 3) who also participated in SAMINOR 1 in 2003–2004. The total number of subjects included was 3671 men and women aged 20–62 years at baseline, of which 1129 were Sami and 2542 non-Sami.

### Questionnaire, blood sample and other measurements

#### Cardiovascular risk factors

We included the following risk factors as outcomes: body mass index (BMI), resting heart rate (RHR), triglycerides, cholesterol, diastolic blood pressure (DBP) and systolic blood pressure (SBP). Change in risk factors is expressed as the difference in values between baseline and follow-up. Non-fasting blood samples were collected and analysed for serum total cholesterol and triglycerides. At both surveys, blood lipids were measured directly by an enzymatic method (Hitachi auto analyser, Roche Diagnostic, Switzerland). These laboratory investigations were performed at the Laboratory of the Department of Clinical Chemistry, University Hospital in Ullevål, Oslo, Norway.

The height and weight of all subjects were recorded. In both studies, RHR, SBP and DBP were measured automatically by the Dinamap (Criticon) blood pressure monitor [16]. Three measurements were taken with an interval of 1 min, and the mean value of the second and third measurements of blood pressure is used in the present analyses. The lowest of the three heart rate measurements was chosen as the RHR value.

#### Leisure time physical activity

In both surveys, leisure time physical activity was assessed by the ‘Saltin-Grimby’ questionnaire at baseline and follow-up [17] and graded 1–4 as follows:

1. Sedentary: Reading, watching TV or other sedentary activities.
2. Moderate: Walking, bicycling, or moving around in other ways at least 4 h a week (including walking or cycling to place of work, walks on Sundays, etc.).
3. Intermediate: Participating in recreational sports, heavy garden work, etc. (note: duration of activity at least 4 h a week).
4. Intensive: Participating in hard training or athletic competitions regularly and several times a week.

To define change in leisure time physical activity, the original variable leisure time physical activity was dichotomized into a *Sedentary* group representing the original sedentary category, with all other categories defined as *Active*. Based on this dichotomized variable, we constructed a new variable labelled ‘Change in physical activity’ with the following categories:

1. Sedentary in both surveys,
2. Reduced activity from active to sedentary,
3. Increased activity from sedentary to active and
4. Active in both surveys.

### Covariates

Age was obtained from the National Population Registry. Daily smoking was self-reported by the question: ‘Are you currently a smoker’ (yes/no)? In SAMINOR 1, ethnicity was measured using the following questions: *What language(s) do/did you, your parents and your grandparents use at home?* The questions were to be answered separately for each relative. The available responses were ‘Norwegian’, ‘Sami’, ‘Kven’ and ‘Other’. The Kvens are subjects whose ancestry can be traced to the Finnish people who immigrated to Northern Norway in the eighteenth century. Multiple answers were allowed for each question. Providing the same response options, we also asked: *What is your, your father’s and your mother’s ethnic background?* The respondents also reported whether they considered themselves to be Norwegian, Sami, Kven or other (self-perceived ethnicity) [15]. In the present study, the Sami population were those who consider themselves to be Sami or reported to have a Sami ethnic background. In addition, at least one grandparent, parents or the participant him/herself should speak Sami language at home to qualify as a Sami. The remaining participants were categorized as non-Sami.

### Statistical analysis

**Figure 1.**
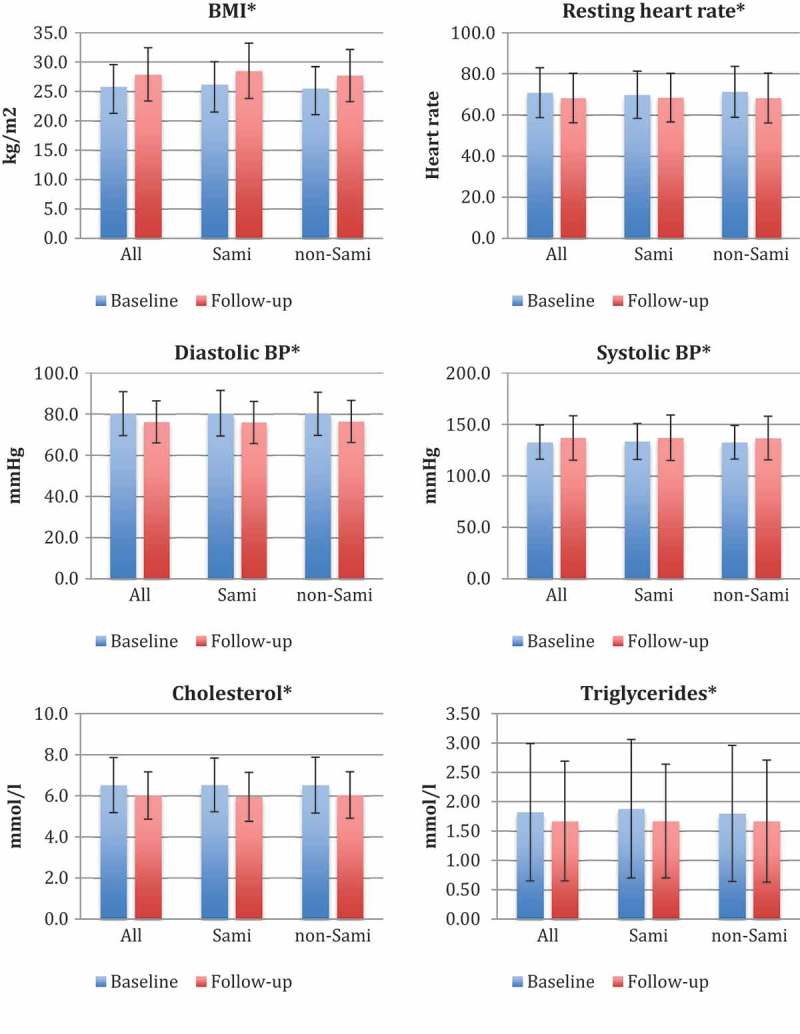
Changes in cardiovascular risk factors (body mass index, resting heart rate, diastolic blood pressure, systolic blood pressure, cholesterol, triglycerides) from 1987/1988 to 2003/2004. ^a^ All differences between baseline and follow-up were significant at *p* < 0.001 for all risk factors. Error bars indicate SD.

Data analyses were performed using IBM SPSS Statistics, version 23 (IBM Corporation, Armonk, NY, USA). A chi-square test for association was conducted to estimate differences in leisure time physical activity between Sami and non-Sami, and paired-samples *t*-test was used to determine whether there were statistically significant changes in unadjusted risk factors from baseline to follow-up (Figure 1). McNemar´s test was applied to test the difference in the proportion of sedentary individuals in the two surveys. ANCOVA analysis was used to test the association between change in leisure time physical activity and change in cardiovascular risk factors. To test any statistical difference between physical activity groups, we used the contrast function in ANCOVA. ANCOVA analyses were adjusted for age, sex, self-reported daily smoking, ethnicity and baseline values. Results were presented as mean differences between baseline and follow-up values with 95% confidence intervals. Possible interactions between physical activity and ethnicity, and between physical activity and sex were assessed by adding multiplicative interaction terms to the main multivariable models. Model assumptions were assessed by visual inspection of residual plots. Triglyceride values did not satisfy model assumptions and were log transformed. *P* values were two-sided with a significance level of 0.05.

## Results

Characteristics of participants at baseline and follow-up by leisure time physical activity level are shown in Tables 1–2. The study included 1886 female and 1785 male participants. Mean baseline age was 45.2 (SD 8.6) years. Sami affiliation was reported by 1129 participants (30.8%). There was a reduction in BMI, DBP, RHR, triglycerides and smoking prevalence across subgroups of leisure time physical activity at baseline (Table 1), which was observed in both ethnic groups (Table 1). At follow-up, BMI, triglycerides, the prevalence of smoking and treatment for hypertension decreased with increasing levels of leisure time physical activity (Table 2). Overall, the proportion of sedentary individuals decreased from 27.8% at baseline to 24.6% at follow-up (*p* < 0.05). In both surveys, a higher proportion of the Samis compared to the non-Samis were sedentary (*p* < 0.05; Table 1–2).

**Table 1.** Characteristics of participants at baseline (Finnmark 3, 1987–1988) by leisure time physical activity level.

		Sami (*n* = 1129)				Non-Sami (*n* = 2542)			
	Total (*n* = 3671)	Sedentary	Moderate activity	Intermediate activity	Intensive activity	Sedentary	Moderate activity	Intermediate activity	Intensive activity
*N* (%)		356 (31.5)	569 (50.4)	182 (16.1)	22 (1.9)	664 (26.1)	1475 (58.0)	371 (14.6)	32 (1.3)
Men (%)	1785 (48.6)	155 (28.9)	229 (42.6)	136 (25.3)	17 (3.2)	327 (26.2)	634 (50.8)	261 (20.9)	26 (2.1)
Age (years)	Mean 45.2 (SD 8.6)	43.6 (8.7)	46.7 (8.6)	44.6 (9.1)	36.4 (9.5)	44.0 (8.3)	46.0 (8.6)	44.7 (8.1)	38.7 (7.6)
Body height (cm)	Mean 166.6 (SD 9.4)	161.6 (8.8)	161.1 (8.3)	166.6 (8.3)	166.2 (7.4)	168.3 (9.1)	167.6 (8.9)	171.7 (8.9)	174.4 (8.4)
Body weight (kg)	Mean 71.6 (SD 12.5)	69.2 (11.8)	68.6 (11.4)	70.5 (9.9)	67.8 (9.7)	73.6 (14.2)	71.8 (12.5)	74.2 (11.4)	75.1 (9.8)
BMI (kg/m^2^)	Mean 25.8 (SD 3.8)	26.5 (4.2)	26.4 (3.9)	25.4 (2.9)	24.5 (2.9)	25.9 (4.3)	25.5 (3.7)	25.1 (2.9)	24.6 (2.3)
Systolic blood pressure (mmHg)	Mean 132.8 (SD 16.7)	133.1 (18.3)	134.4 (17.5)	131.5 (15.6)	125.8 (12.0)	132.6 (16.1)	132.4 (16.4)	133.0 (16.6)	135.3 (14.7)
Diastolic blood pressure (mmHg)	Mean 80.2 (SD 10.7)	80.7 (11.4)	81.2 (10.7)	78.2 (11.0)	72.1 (9.7)	80.7 (10.5)	79.9 (10.3)	80.0 (11.1)	76.5 (12.4)
RHR (beats/min)	Mean 70.8 (SD 12.1)	71.8 (10.5)	70.4 (11.4)	65.5 (11.4)	57.5 (12.8)	73.6 (11.8)	71.6 (12.3)	66.6 (11.8)	56.2 (9.8)
Cholesterol (mmol/l)	Mean 6.52 (SD 1.34)	6.47 (1.29)	6.59 (1.30)	6.56 (1.34)	5.46 (0.90)	6.56 (1.41)	6.56 (1.33)	6.38 (1.33)	5.70 (1.24)
Triglycerides (mmol/l)	Mean 1.82 (SD 1.17)	2.00 (1.29)	1.84 (1.16)	1.83 (1.06)	1.30 (0.60)	1.93 (1.14)	1.77 (1.21)	1.69 (0.97)	1.54 (0.92)
Smokers % (*n*)	41.1 (1507)	48.0 (171)	35.7 (203)	38.5 (70)	18.2 [4]	52.7 (350)	39.3 (579)	34.8 (129)	3.1 [1]
Treat hypertension % (*n*)	5.6 (206)	3.9 [14]	6.5 [37]	3.3 [6]	0.0 (0)	6.5 [43]	6.1 (90)	3.8 [14]	6.3 [2]

Data are presented as mean (SD) or % (*n*). BMI: body mass index; RHR: resting heart rate

**Table 2.** Characteristics of participants at follow-up (SAMINOR 1, 2003–2004) by leisure time physical activity level.

		Sami (*n* = 1129)				Non-Sami (*n* = 2542)			
	Total (*n* = 3671)	Sedentary	Moderate activity	Intermediate activity	Intensive activity	Sedentary	Moderate activity	Intermediate activity	Intensive activity
*N* (%)		310 (27.5)	676 (59.9)	132 (11.7)	11 (1.0)	593 (23.3)	1548 (60.9)	383 (15.1)	18 (0.7)
Men (%)	1785 (48.6)	134 (25.0)	304 (56.6)	90 (16.8)	9 (1.7)	274 (22.0)	726 (58.2)	231 (18.5)	17 (1.4)
Age (years)	Mean 61.2 (SD 8.6)	61.1 (9.5)	61.4 (8.8)	60.6 (8.7)	58.6 (6.2)	60.5 (9.4)	61.6 (8.1)	60.9 (8.5)	57.2 (9.2)
Body height (cm)	Mean 165.3 (SD 9.5)	160.2 (9.2)	160.6 (8.4)	164.2 (9.0)	164.1 (9.7)	166.2 (9.3)	166.9 (9.1)	169.5 (9.2)	175.2 (9.5)
Body weight (kg)	Mean 76.4 (SD 14.3)	74.9 (14.2)	73.3 (13.5)	74.6 (12.1)	69.0 (6.6)	78.8 (16.0)	76.8 (14.0)	78.3 (13.9)	79.0 (9.6)
BMI (kg/m^2^)	Mean 27.9 (SD 4.5)	29.2 (5.4)	28.4 (4.5)	27.7 (4.0)	25.7 (1.6)	28.5 (5.3)	27.5 (4.2)	27.2 (3.9)	25.7 (1.5)
Systolic blood pressure (mmHg)	Mean 136.8 (SD 21.5)	137.6 (22.6)	136.7 (22.2)	137.7 (20.6)	133.4 (23.8)	137.8 (22.0)	136.7 (21.3)	135.4 (20.0)	129.9 (13.2)
Diastolic blood pressure (mmHg)	Mean 76.2 (SD 10.2)	74.9 (10.1)	75.8 (10.4)	78.1 (9.4)	76.6 (9.2)	76.4 (10.6)	76.4 (10.1)	76.0 (10.2)	75.6 (9.2)
RHR (beats/min)	Mean 68.2 (SD 12.0)	69.0 (12.5)	68.4 (11.4)	67.6 (12.2)	61.2 (15.0)	69.5 (12.7)	68.3 (11.8)	66.1 (11.9)	55.6 (9.4)
Cholesterol (mmol/l)	Mean 6.01 (SD 1.15)	5.98 (1.16)	5.93 (1.18)	5.96 (1.33)	6.14 (1.30)	6.00 (1.13)	6.04 (1.13)	6.07 (1.09)	6.09 (0.97)
Triglycerides (mmol/l)	Mean 1.67 (SD 1.02)	1.74 (0.98)	1.67 (0.94)	1.57 (1.08)	1.37 (0.70)	1.85 (1.38)	1.62 (0.90)	1.58 (0.93)	1.26 (0.54)
Smokers % (*n*)	28.3 (1039)	31.2 (96)	27.5 (184)	22.9 [30]	0.0 (0)	35.4 (209)	26.3 (404)	30.0 (114)	11.1 [2]
Treat hypertension % (*n*)	27.0 (991)	32.0 (99)	28.1 (187)	20.0 [26]	30 [3]	31.5 (185)	26.6 (410)	21.3 (81)	0.0 (0)

Data are presented as mean (SD) or % (*n*). BMI: body mass index; RHR: resting heart rate

All cardiovascular risk factors in the total cohort changed over time, with an increase in BMI with 2.2 kg/m^2^ and SBP with 4.0 mmHg, and a decrease in RHR with 2.5 beats/min, DBP with 4.0 mmHg, cholesterol with 0.51 mmol/l and triglyceride levels with 0.15 mmol/l (*p* < 0.001; Figure 1). These relationships were consistent irrespective of ethnicity.

After adjustments for age, sex, smoking habits, ethnicity and respective baseline values, there were statistically significant differences between persistent physical activity vs persistent sedentary for BMI (*p* = 0.035) and cholesterol (*p* = 0.003) and between persistent active and active to sedentary group for triglycerides (*p* = 0.005) (Table 3). Those who were active during both surveys had a 0.3 kg/m^2^ lower increase in BMI compared to those who were sedentary in both surveys (*p* = 0.025). On the other hand, those who were persistently active had the lowest reduction in cholesterol compared to the persistently sedentary group (*p* = 0.003). RHR, diastolic and systolic blood pressure did not change significantly with change in physical activity. Further adjustment for anti-hypertensive medication at follow-up did not change the results significantly for diastolic and systolic blood pressure.

**Table 3.** Associations between change in leisure time physical activity and change in risk factors between baseline (Finnmark 3, 1987–1988) and follow-up (SAMINOR 1, 2003–2004).

	Sedentary in both	Active to sedentary	Sedentary to active	Active in both	
*n*	422	481	598	2170	*P equality*
BMI (kg/m^2^)	2.4 (2.2, 2.7)	2.4 (2.2, 2.6)	2.2 (2.0, 2.4)	2.1 (2.0, 2.2)	0.035
Resting heart rate (beats/min)	−2.3 (−3.3, −1.3)	−2.1 (−3.1, −1.2)	−2.9 (−3.7, −2.0)	−2.6 (−3.0, −2.1)	0.665
Triglycerides (%)^a^	−4.2 (−8.2, −0.1)	−0.8 (−4.6, 3.2)	−8.9 (−12.0, −5.5)	−7.3 (−9.1, −5.6)	0.005
Cholesterol (mmol/l)	−0.64 (−0.74, −0.53)	−0.51 (−0.60, −0.41)	−0.60 (−0.69, −0.52)	−0.46 (−0.51, −0.42)	0.003
Diastolic blood pressure (mmHg) ^b^	−4.7 (−5.6, −3.9)	−4.1 (−4.9, −3.3)	−4.3 (−5.0, −3.5)	−3.7 (−4.1, −3.3)	0.133
Systolic blood pressure (mmHg) ^b^	4.7 (3.0, 6.4)	4.2 (2.6, 5.8)	3.7 (2.3, 5.2)	3.9 (3.1, 4.6)	0.811

Data are presented as mean change (95% CI) and adjusted for age, smoking habits, sex, ethnicity and baseline values. BMI: body mass index ^a^ Triglyceride values were log transformed and presented as change in percent; ^b^ Adjusted for anti-hypertensive medication at follow-up.

There were no statistically significant interactions (*p* > 0.05) between ethnicity and change in leisure time physical activity, except for cholesterol, which changed significantly with physical activity in the non-Sami, but not in the Sami group (*p* for interaction = 0.014). Furthermore, there were no statistically significant interaction between sex and change in leisure time physical activity.

## Discussion

In this longitudinal, population-based study over 16 years, we found a lower increase in BMI among those who were active during both surveys compared to those who were persistently sedentary in leisure time. Furthermore, we found that the decrease in triglycerides was more pronounced in the persistently active group than in the persistently sedentary participants. To our surprise, those who were persistently active had the lowest reduction in cholesterol. Otherwise, changes in leisure time physical activity were not reflected in the changes in cardiovascular risk factors of clinical significance. A higher proportion of the Samis compared to non-Samis were sedentary in leisure time; however, ethnicity did not influence the association between leisure time physical activity and cardiovascular risk factors. Our study extends existing knowledge on physical activity and cardiovascular risk factors by exploiting repeated measurements of both physical activity and risk factors.

In general, we found little impact of change in physical activity on change in risk factors, which is somewhat surprising considering the abundance of studies suggesting beneficial effects of physical activity on cardiovascular risk factors and CVD. An explanation may be that the physical activity level and changes are too modest to initiate beneficial effect on the measured risk factors.

We found an increase in BMI over the follow-up period, in accordance with a global trend of increasing BMI over decades, including northern Norway [18,19], which raises concerns considering the relationship with diabetes, cardiovascular diseases and all-cause mortality [20–23]. As expected, we found that BMI increased most among those who were persistently inactive. Anderssen et al. [24] explored the development of BMI from 1972 to 2002 in a Norwegian cohort and found, in accordance with our study, a higher prevalence of obesity among the sedentary compared to people with higher levels of physical activity.

In contrast to the increase in BMI and SBP during follow-up, we found a decrease in RHR, DBP, cholesterol and triglyceride levels. This may be explained by more frequent use of anti-hypertensive medication, statins, etc. at follow-up. Results from this study show an increase in anti-hypertensive medication from about 5% at baseline to nearly 28% at follow-up. However, adjustment for anti-hypertensive medication did not influence the results. We do not have information about any changes in the use of statins, but it seems likely that the use of statins has increased during follow-up as well.

RHR is affected by numerous factors, including genetics and physical fitness [25], and engaging in regular physical activity is associated with lower RHR [26]. In the present study, RHR decreased over time, but the decrease was not associated with change in leisure time physical activity levels. The decrease in RHR from 1987 to 2003–2004 shown in the present study is in accordance with results from the Tromsø Study (1986–2007) [27]. Sharashova et al. [27] found a considerable decline in RHR for men and women, and this development was partly related to changes in cardiovascular risk factors such as SBP, triglycerides, increased physical activity, blood pressure medication and smoking cessation.

Present knowledge supports that physical activity and exercise have a positive impact on lipids, although often a small effect [28]. This is consistent with the present study, where triglycerides decreased most among those who increased their activity level or were persistently active. Nevertheless, an active lifestyle is recommended to patients with abnormal lipids [29].

We found a reduction in DBP over time, while SBP, on the other hand, increased. This is in accordance with data from NHANES III [30], showing that average SBP in the US population increased throughout life, while DBP decreased after 60 years of age. The American College of Sports Medicine recommends dynamic aerobic endurance training for at least 30 min daily to prevent hypertension [31], and randomized control studies repeatedly show that exercise reduces SBP and DBP [32]. There seems to be strong evidence for an effect of physical activity interventions on blood pressure not only among hypertensive but also among normotensive and prehypertensive individuals [33]. However, in our study we found no association between change in leisure time physical activity and blood pressure.

We have previously shown that Sami men and women in the 1980s were more physically active at work and thus had a higher total physical activity score than Norse men and women [34]. In the present study, a higher proportion of the Sami compared to non-Sami were inactive in leisure time in both surveys. However, we do not have comparable measures on occupational physical activity at follow-up, and the results from this study are solely based on leisure time physical activity, making it hard to draw conclusions on trends in total physical activity. The traditional Sami way of living, with a major engagement in the primary industries, has changed over the last decades, the Sami society is modernized and mechanization has most likely entailed less occupational physical activity. From 1980 to 2000, the prevalence of Samis working in primary industry decreased from 20% to 12% [35]. Thus, one would expect that the total physical activity level among Samis gradually would approach the level of the non-Samis. At follow-up, there were no differences in RHR between Sami and non-Sami, supporting the assumption that the level of physical activity between the two groups over time have become more comparable. The same trend with Westernization and urbanization is seen among the Canadian and Greenland Inuit; since 1950, a gradual transition from a traditional lifestyle characterized of hunting and fishing to a more sedate lifestyle with reduced physical activity and less favourable cardiovascular risk factors was observed [36,37].

This study has limitations. Even with a participation rate of 78% in Finnmark 3 and 63% in SAMINOR 1, individuals with poor health were presumably underrepresented, introducing potential selection bias. However, bias due to non-participation is probably of less concern in prospective studies [38,39], because the outcome is not known at baseline and will not affect the way participants respond to the questionnaire. Moreover, the use of self-reported physical activity has most likely introduced misclassification errors. Given that people tend to overestimate their activity level [40], misclassification will probably underestimate the real effects of physical activity [41]. The validity of our physical activity questions has been ensured in several studies [40,42], and physical activity according to the questionnaire was found to be positively associated with objectively measured physical activity and physical fitness in a dose–response relationship [40].

Moreover, lack of information about medications such as β-blockers and lipid lowering drugs could bias our results, especially if the prevalence of these drugs were different in ethnic groups. However, Sami and non-Sami in this study have a very similar cardiovascular risk profile both at baseline and follow-up and we have no clear indications that there is a skewness between these groups in medication.

Pettersen et al. [43] have challenged the concept of ethnicity, which is a complex topic. The inclusion criteria and definition have impacts on the size of the study population. Different definitions of ethnicity were used in the Finnmark 3 Study and SAMNIOR 1; the latter has somewhat broader definition and is now widely used, and was therefore the chosen definition in the present study.

This cohort study with a prospective design and 16 years of follow-up has the advantage of being one of few studies with repeated measurements of both leisure time physical activity and cardiovascular risk factors. Furthermore, this is to our knowledge the first study highlighting this topic of association between changes in leisure time physical activity and cardiovascular risk factors in the Arctic region of Norway.

## Conclusions

In this longitudinal cohort study, being persistently physically active over a 16-year period was reflected in a lower increase in BMI than being sedentary. In addition, the decrease in triglyceride levels was more pronounced in the persistently active group than in the persistently sedentary group. Otherwise, the impact of change in physical activity on cardiovascular risk factors was minor and clinically insignificant. The Sami participants were more sedentary than the non-Sami; however, there were few ethnic differences in the association between leisure time physical activity and cardiovascular risk factors.
